# The sleep patterns and their associations with mental health among nursing home residents: a latent profile approach

**DOI:** 10.1186/s12877-023-04124-5

**Published:** 2023-08-03

**Authors:** Huanyu Mou, Dongjuan Xu, Shanshan Zhu, Meng Zhao, Yaqi Wang, Kefang Wang

**Affiliations:** 1 School of Nursing and Rehabilitation, Cheeloo College of Medicine, Shandong University, Jinan, Shandong Province 250012 China; 2 The Nethersole School of Nursing, Faculty of Medicine, The Chinese University of Hong Kong, Shatin, N.T., Hong Kong SAR China; 3 School of Nursing, Purdue University, West Lafayette, IN 47907 USA; 4 Geriatrics Department, Henan Provincial People’s Hospital, Zhengzhou, Henan Province 450000 China

**Keywords:** Sleep, Latent profile analysis, Depression, Anxiety, Nursing homes

## Abstract

**Background:**

Nursing home residents commonly experience poor sleep conditions. However, few studies have explored the potential sleep patterns among nursing home residents. This study aimed to identify the sleep patterns in nursing home residents, compare residents’ characteristics across sleep patterns, and examine the relationships between sleep patterns and residents’ mental health (i.e., depressive and anxiety symptoms).

**Methods:**

This cross-sectional study was conducted in 27 nursing homes in Jinan, China, from March to June 2018. In total, 353 participants were recruited via convenience sampling, and of which, 326 completed the survey. A latent profile analysis was performed to identify sleep patterns based on the seven dimensions of the Pittsburgh Sleep Quality Index. Bivariate analyses were conducted to compare residents’ characteristics among the sleep patterns. Mixed-effects logistic regression analyses were adopted to investigate the relationships between sleep patterns and residents’ mental health.

**Results:**

Three sleep patterns were identified, including ‘good sleepers’, ‘poor sleepers without hypnotic use’, and ‘poor sleepers with hypnotic use’. Residents’ gender, education, pain, instrumental activities of daily living, and number of chronic conditions were significantly differentiated across the sleep patterns. Compared with ‘good sleepers’, ‘poor sleepers without hypnotic use’ were significantly associated with more depressive symptoms (OR = 3.73, 95% CI = 2.09, 6.65, *p* < 0.001), but not with anxiety symptoms (OR = 2.04, 95% CI = 0.97, 4.29, *p* = 0.062); whereas ‘poor sleepers with hypnotic use’ had significantly more depressive (OR = 5.24, 95% CI = 2.54, 10.79, *p* < 0.001) and anxiety symptoms (OR = 5.02, 95% CI = 2.13, 11.83, *p* < 0.001).

**Conclusions:**

This study reveals three distinct sleep patterns in nursing home residents and their significant associations with residents’ mental health. These findings can inform future research to develop appropriate and tailored intervention strategies for improving sleep and promoting mental health for nursing home residents.

**Supplementary Information:**

The online version contains supplementary material available at 10.1186/s12877-023-04124-5.

## Introduction

Nursing home residents commonly experience poor sleep conditions. As reported by recent literature, 20–73% of residents in long-term care facilities had sleep disturbances, which was higher than those living in the community, probably due to institutional residents’ vulnerable health conditions, poor sleep hygiene, inappropriate light exposure, and nighttime noise in the institutional environments [1, 2]. Therefore, it is imperative to further explore and deeply understand sleep conditions and patterns to promote sleep management for nursing home residents.

Sleep patterns can be characterized by multiple sleep dimensions, such as sleep onset latency, duration, efficiency, and daytime napping. Generally, nursing home residents experience segmented sleep, characterized by low sleep efficiency, excessive time spent in bed, and increased wake time during the night [2, 3]. In terms of quality, more than 70% of older adults in long-term care facilities had poor sleep quality [2]. More importantly, some older adults have co-occurring sleep complaints and problems (e.g., prolonged sleep latency and shortened sleep duration), meaning that they probably experience distinct sleep patterns with different combinations of problems in these sleep dimensions [1, 4]. Therefore, instead of only observing the overall sleep condition or single sleep dimension, exploring sleep patterns by considering the combined effects and complex relationships of multiple sleep dimensions may help recognize the potential subgroups of residents with similar sleep characteristics, and thus to help achieve the targeted and individualized sleep management.

To date, several studies have identified different sleep patterns based on the sleep dimensions (e.g., sleep duration, efficiency) in samples of healthy adults [5], chronic insomnia adults [6], older women [7], and community-dwelling older adults [4, 8]. For instance, Yu and colleagues (2017) detected four sleep patterns of ‘inadequate sleep’, ‘disturbed sleep’, ‘trouble falling asleep’, and ‘multiple problems’ in the sample of 230 older individuals with sleep problems [4]. Chen and colleagues suggested four sleep patterns for community-dwelling older adults, i.e., ‘high hypnotics’, ‘high insomnia’, ‘mild insomnia’ and ‘fair sleep’ [8]. In Leigh et al.’s (2015) study, older women were grouped into four sleep patterns, including ‘troubled sleepers’, ‘early wakers’, ‘troubling falling sleep’, and ‘untroubled sleepers’ [7]. However, few studies provide insight into the potential sleep patterns in nursing home residents. Therefore, it is indeed necessary to undertake such research in nursing home residents, which has important implications for developing tailored intervention strategies based on the different sleep patterns to improve residents’ sleep quality.

Moreover, to get a complete picture of sleep conditions, it is crucial to be inclusive of all sleep dimensions. Currently, sleep duration, onset latency, sleep efficiency, occurrence of sleep disturbances, and daytime dysfunction are commonly used dimensions for identifying sleep patterns [4, 5, 7, 8]. On top of these aforementioned dimensions, the use of sleep medication was only considered in several studies focusing on community-dwelling older adults or people with chronic insomnia [6, 8]. Previous studies suggested that approximately 50% of nursing home residents reported regular utilization of hypnotic drugs [9, 10]. Considering the high prevalence of sleep medication use in nursing home residents, it is particularly significant to include the use of hypnotics in identifying sleep patterns. The Pittsburgh Sleep Quality Index (PSQI) with seven dimensions including hypnotic use serves as a great tool for sleep pattern studies [5, 8].

Previous studies have suggested a potential causal relationship between sleep disturbance and mental health among older adults [11–14]. Longitudinal studies have demonstrated that older adults with disturbed sleep experienced an increased risk of developing anxiety and depressive symptoms, compared with those without sleep disturbance [13, 14]. Several randomized controlled trials have also suggested that sleep interventions (e.g., cognitive behavior therapy for insomnia) were effective in reducing depression among older adults [15, 16]. Different sleep patterns in older adults appear to demonstrate distinct associations with mental health. In a recent meta-analysis [17], older adults with a lack of good sleep quality were significantly associated with depression. In Chen et al. (2020) study [8], the sleep patterns of ‘high hypnotics’, ‘high insomnia’ and ‘mild insomnia’ were associated with more severe depression and anxiety symptoms compared with those with ‘fair sleep quality’. However, little is known about the relationships between nursing home residents’ sleep patterns and depression/anxiety symptoms.

Using a sample of nursing home residents, this study aimed to (1) identify nursing home residents’ sleep patterns via the Latent Profile Analysis (LPA) as indexed by the seven sleep dimensions of the PSQI, (2) compare residents’ characteristics across sleep patterns, and (3) examine the relationships between sleep patterns and residents’ mental health (i.e., depressive and anxiety symptoms).

## Methods

### Study design and participants

A cross-sectional study was conducted from March to June 2018 in the nursing homes of Jinan, Shandong Province, China. Nursing homes that had at least 30 beds and had operated for at least 1 year were invited to participate. According to the registration on the Jinan Civil Affairs Bureau website, there were 69 eligible nursing homes in the five districts (Huaiyin, Licheng, Lixia, Shizhong, and Tianqiao) of Jinan. Of these, 42 nursing homes were excluded due to refusal to participate (n = 28), contact but no response (n = 8), and renovation (n = 6). Therefore, residents were recruited from 27 nursing homes using a convenience sampling method. Researchers first contacted facility administrators and obtained their permission to conduct the survey.

For residents, the inclusion criteria were (1) aged 60 years or above, (2) having lived in nursing homes for 3 months or longer, and (3) being willing and able to participate in this study. The residents were excluded if they had (1) severe cognitive impairment, as indicated by the score of Mini-Mental State Examination (MMSE) < 10 [18]; (2) vision or hearing impairment, preventing the completion of data collection; and (3) coma, end-stage disease, or hospice care. Previous studies have shown that persons with mild to moderate cognitive impairment are able to respond consistently to standardized questionnaire items and articulate feelings, providing reliable responses to questions about their health and quality of life [19, 20].

The study was approved by the Shandong University Institutional Review Board (Ref. 2017-R-112). Research assistants attended training sessions to go over research ethics, informed consent, study design, data collection procedure, and each item in the questionnaire. Before data collection, written informed consent was obtained from residents, and they were assured that all their responses would be kept confidential and anonymous. Research assistants assisted residents to complete the survey through face-to-face interviews. Residents mainly completed the survey on their own. If necessary, research assistants read the questions in the survey and recorded residents’ answers. At the end of the survey, research assistants checked the questionnaire for missing responses.

Among the 27 nursing homes, 1,885 residents were approached to assess their eligibility. A total of 1,532 of them were excluded because of the restrictions of physical functioning such as severe cognitive impairment or hearing loss (n = 1,187), living for less than 3 months (n = 128), age of less than 60 years (n = 59), refusing participation (n = 96), and not residing in nursing homes over the study period (n = 62). A total of 353 eligible residents participated in the survey.

### Measures

#### PSQI

The 19-item self-reported PSQI was employed to evaluate residents’ overall sleep from seven dimensions: subjective sleep quality, sleep latency, sleep duration, sleep efficiency, sleep disturbances, sleep medicine use, and daytime dysfunction [21]. The total score ranged from 0 to 21, with each component scored 0 to 3. Higher scores represented poorer sleep, with a global score over 7 suggestive of poor overall sleep [22]. The Chinese-version PSQI has been translated and validated with satisfactory reliability and validity [22]. The Cronbach’s alpha coefficient in this study was 0.78.

#### Mental health

Depressive and anxiety symptoms were screened using the Patient Health Questionnaire-9 (PHQ-9) and Generalized Anxiety Disorder Scale-2 (GAD-2), respectively. The PHQ-9 covers nine signs and symptoms of depression derived from the Diagnostic and Statistical Manual of Mental Disorders, Fourth Edition [23]. The sum scores ranged from 0 to 27. Higher scores suggested more severe depressive symptoms, with a cut-off score of 5 or above indicative of depressive symptoms [24]. The GAD-2 was a two-item self-reported questionnaire for screening anxiety symptoms in the past two weeks [25]. Each item was responded to on a 4-point scale (i.e., 0 to 3). A total score of 3 or greater indicated anxiety symptoms [26]. In the present study, the Cronbach’s alpha coefficients of PHQ-9 and GAD-2 were 0.81 and 0.71, respectively.

#### Control variables

Residents’ demographic characteristics included gender, age, education, living room, and length of stay. The health-related characteristics consisted of pain, activities of daily living (ADLs), instrumental activities of daily living (IADLs), cognitive function, and number of chronic conditions. The performance in ADLs was measured by the Barthel Index, which contained the following ten activities including bathing, grooming, feeding, dressing, bladder management, bowel management, toilet use, ambulation, transferring, and stair climbing [27]. Residents’ independence in IADLs was determined by their performance in doing light housework, using the telephone, and taking the right medicine at the right time [28]. Residents were considered ADLs or IADLs independent if they reported full ability to perform each included task; otherwise, they were regarded as ADLs or IADLs dependent [28]. The number of chronic conditions was assessed according to the 16 clinical conditions derived from the Functional Comorbidity Index (FCI) [29], in which depression and anxiety were excluded from the calculation. The presence of each condition was scored 1 and the absence was 0, with total scores ranging from 0 to 16. Cognitive function was evaluated by MMSE [30]. The MMSE adopted a set of 11 questions and the total score ranges from 0 to 30. Residents’ cognitive impairment could be categorized into four levels: no (27–30), mild (21–26), moderate (10–20), and severe impairment (0–9) [31]. Information on nursing home characteristics was also collected, including ownership, size (total beds), affiliation, occupation rate, staff-resident ratio, staff working hours per day, and the availability of outdoor spaces.

#### Statistical analyses

LPA was conducted to identify the sleep patterns (i.e., latent classes) experienced by nursing home residents. LPA is a data-driven statistical approach that directly derives the potential classes from the original dataset without priori hypotheses, which can help differentiate the existing sleep patterns by clustering homogeneous responses on sleep dimensions [32]. The goal was to allocate residents into distinct sleep patterns based on the scores in the seven sleep dimensions of the PSQI, so that residents within a certain pattern had more similar sleep characteristics than those in other patterns. According to the previous literature that examined statistical power to detect the number of latent classes in latent profile analysis [33], a sample size of 250 with a very large degree of separation (Cohen’s *d* = 1.5) was acceptable to capture the potential classes based on the statistical indices: Akaike’s Information Criterion (AIC), Bayesian Information Criterion (BIC), Entropy, Lo-Mendell-Rubin Likelihood Ratio Test (LMRT), and Bootstrapped-Likelihood Ratio Test (BLRT), as well as the theoretical justification and interpretability. The values of AIC and BIC reflected the goodness-of-fit and parsimony of the identified model, with smaller values indicating superior fitting modeling [34]. Higher entropy value pointed to greater accuracy of the classification [33]. In addition, LMRT and BLRT, as the inferential tests, suggested whether the model of the k-class had a better fit than that of the k-1 class [33]. The model was begun by specifying a 2-class model, and then was gradually expanded by including additional classes, until arriving at the optimal classification that could achieve the best fitting model and the meaningful interpretation of clinical practice. Moreover, the additional class would be rejected if it included few than 50 cases or 5% of the total sample size [35].

After the optimal sleep patterns were determined, the one-way ANOVA analyses were conducted to compare the differences across sleep patterns in terms of the seven sleep dimensions. Bivariate analyses (e.g., Chi-square test, one-way ANOVA, and Kruskal-Wallis test) were conducted to compare residents’ demographic and health characteristics among sleep patterns. Finally, the mixed-effects logistic regression analyses were performed to investigate the relationships between sleep patterns and residents’ mental health (i.e., depressive and anxiety symptoms), in which odds ratios (ORs) and 95% confidence intervals (CIs) were calculated. The resident-level (i.e., gender, age, education, living room, length of stay, pain, ADLs, IADLs, FCI, and MMSE) and facility-level (i.e., ownership, size, affiliation, occupation rate, staff-resident ratio, staff working hours per day, and outdoor sites) variables were controlled in the adjusted models. LPA was performed using the Mplus Version 7 (Muthén & Muthén, Los Angeles, CA). All descriptive statistics, bivariate analyses, and regression were conducted via Stata version 14.1 (Stata Corp, College Station, TX), with *p* values < 0.05 indicating statistical significance.

## Results

### Participants’ characteristics

Among the 353 eligible nursing home residents, 27 residents could not comprehend questions well or remember their sleep issues correctly and were excluded from the data analysis. A total of 326 residents from 27 nursing homes were included in the present study. Table 1 presents the characteristics of the residents and nursing homes. The mean age of residents was 78.81 ± 8.90 years, 54.6% were female, and most (82.5%) lived in shared rooms. The prevalence of poor sleep was high, with 62.6% of residents reporting poor overall sleep. For mental health, almost 40% of residents reported depressive symptoms and approximately 18% of them experienced anxiety symptoms.

**Table 1:** Characteristics of the residents and nursing homes

Variables	M ± SD (n%)
***Resident-level variables (n = 326)***	
Gender	
Male	148 (45.4%)
Female	178 (54.6%)
Age	78.81 ± 8.90
Education	
Illiterate	79 (24.2%)
Elementary/middle school	166 (50.9%)
High school or above	81 (24.8%)
Living room	
Single/couple	57 (17.5%)
Shared	269 (82.5%)
Length of stay	
≤ 1 year	125 (38.3%)
1–3 years	116 (35.6%)
> 3 years	85 (26.0%)
Pain	
Yes	143 (43.9%)
No	183 (56.1%)
Number of chronic conditions (FCI)	2.00 (2.00)^#^
ADLs	
Independent	93 (28.5%)
Dependent	233 (71.5%)
IADLs	
Independent	127 (39%)
Dependent	199 (61%)
Cognitive function (MMSE)	21.52 ± 5.11
No impairment	65 (19.9%)
Mild impairment	135 (41.4%)
Moderate impairment	126 (38.7%)
Sleep (PSQI scores)	9.48 ± 4.76
Subjective sleep quality	1.29 ± 0.89
Sleep latency	1.91 ± 1.24
Sleep duration	1.43 ± 1.22
Sleep efficiency	1.88 ± 1.20
Sleep disturbances	1.10 ± 0.53
Use of sleep medication	0.51 ± 1.10
Daytime dysfunction	1.37 ± 0.84
Overall sleep (PSQI, cutoff of 7)	
Good	122 (37.4%)
Poor	204 (62.6%)
Depressive symptoms (PHQ-9)	
Yes	129 (39.6%)
No	197 (60.4%)
Anxiety symptoms (GAD-2)	
Yes	57 (17.5%)
No	269 (82.5%)
***Nursing home variables (n = 27)***	
Ownership	
Not-for-profit	25 (92.6%)
For-profit	2 (7.4%)
Size (total beds)	100 (65)^#^
Affiliation	
Hospital-based	3 (11.1%)
Freestanding	24 (88.9%)
Occupation rate	0.68 ± 0.23
Staff-resident ratio	5.74 ± 2.21
Staff working hours/week	84.00 (4.00)^#^
Outdoor sites	
Provided	19 (70.4%)
None	8 (29.6%)

Notes: 1) ADLs, Activities of Daily Living; FCI, Functional Comorbidity Index; GAD-2, Generalized Anxiety Disorder Scale-2; IADLs, Instrumental Activities of Daily Living; MMSE, Mini-Mental State Examination; PHQ-9, Patient Health Questionnaire-9; PSQI, Pittsburgh Sleep Quality Index;

2) ^#^: median and interquartile range.

### Sleep patterns in nursing home residents

According to the fit statistics derived from LPA (Table 2), the 3-class model demonstrated the optimal goodness-of-fit for sleep patterns, with relatively low AIC and BIC values (much lower than those in the 2-class model), and a high entropy value (i.e., 0.93). The LMRT values also indicated that the 3-class model had a significantly better fit than the 2-class model (*p* < 0.001), whereas the 4-class model was not better than the 3-class model (*p* = 0.088). Besides, the latent classes in the 3-class model showed a very large degree of separation (Cohen’s *d* = 2.4). Therefore, the 3-class model had a good fit overall and was the most parsimonious and readily interpretable.

**Table 2:** Goodness-of-fit statistics of models (n = 326)

Number of latent classes	Loglikelihood	AIC	BIC	Entropy	LMRT	BLRT	Class probability
1-class model	-3155.50	6338.99	6392.01	—	—	—	—
2-class model	-2660.03	5364.06	5447.37	1.000	0.049	< 0.001	0.83 0.17
3-class model^*^	-2411.36	4882.71	4996.32	0.93	< 0.001	< 0.001	0.40 0.44 0.17
4-class model	-2350.13	4776.27	4920.17	0.93	0.088	< 0.001	0.35 0.17 0.15 0.33
5-class model	-2297.64	4687.29	4861.48	0.94	0.074	< 0.001	0.16 0.28 0.09 0.30 0.17

Notes: 1) AIC, Akaike Information Criterion; BIC, Bayesian Information Criterion; LMRT, Lo-Mendell-Rubin Adjusted Likelihood Test; BLRT, Bootstrap Likelihood Ratio Test.

2) ^*^: Selected model.

**Fig. 1:**
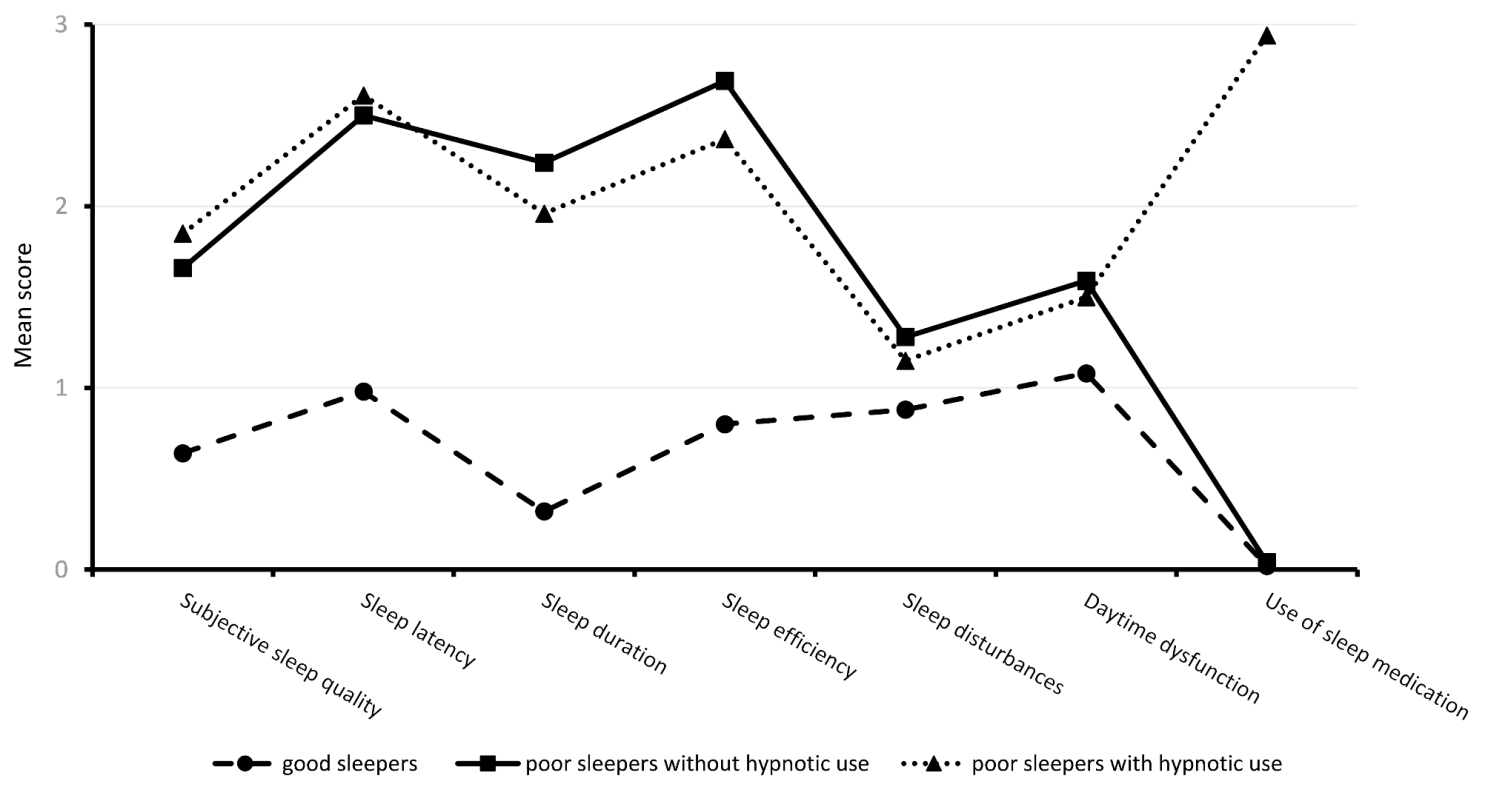
Profile of the sleep patterns in nursing home residents

As shown in Fig. 1; Table 3, there were substantial differences in the seven sleep dimensions across the three classes (*p* < 0.001). Overall, residents in Class 1 reported lower scores in the seven dimensions than those in Classes 2 and 3. Moreover, residents in Classes 2 and 3 had similarly higher scores in these sleep dimensions except for sleep medication use. Residents in Class 3 had a significantly higher score in the dimension of sleep medication use than those in Class 2. As a result, the three sleep patterns in this study were identified as good sleepers (Class 1, 39.9%), poor sleepers without hypnotic use (Class 2, 43.5%), and poor sleepers with hypnotic use (Class 3, 16.6%).

**Table 3:** The characteristics of participants’ sleep patterns (n = 326)

	C1 (n = 130)	C2 (n = 142)	C3 (n = 54)	Statistics
**PSQI**	4.71 ± 2.41	11.99 ± 2.45	14.39 ± 3.38	F = 378.57, *p* = 0.001 (1 < 2 < 3)
Subjective sleep quality	0.64 ± 0.06	1.66 ± 0.08	1.85 ± 0.11	F = 87.07, *p* < 0.001 (1 < 2 and 3)
Sleep latency	0.98 ± 0.15	2.50 ± 0.11	2.61 ± 0.12	F = 94.40, *p* < 0.001 (1 < 2 and 3)
Sleep duration	0.32 ± 0.07	2.24 ± 0.13	1.96 ± 0.14	F = 240.69, *p* < 0.001 (1 < 3 < 2)
Sleep efficiency	0.80 ± 0.12	2.69 ± 0.07	2.37 ± 0.14	F = 214.19, *p* < 0.001 (1 < 3 < 2)
Sleep disturbances	0.88 ± 0.05	1.28 ± 0.05	1.15 ± 0.07	F = 22.89, *p* < 0.001 (1 < 2 and 3)
Use of sleep medication	0.02 ± 0.91	0.04 ± 0.02	2.94 ± 0.03	F = 6439.63, *p* < 0.001 (1 and 2 < 3)
Daytime dysfunction	1.08 ± 0.09	1.59 ± 0.08	1.50 ± 0.10	F = 10.68, *p* < 0.001 (1 < 2 = 3)
**Socio-demographic characteristics**				
Gender (% female)	59 (45.4%)	80 (56.3%)	39 (72.2%)	χ^2^ = 11.39, *p* = 0.003 (1 and 2 < 3)
Age	77.42 ± 9.53	79.42 ± 8.60	80.54 ± 7.68	F = 2.99, *p* = 0.052
Education				KW = 11.55, *p* = 0.003 (2 < 1)
Illiterate	23 (17.7%)	43 (30.3%)	13(24.1%)	
Elementary/middle school	63 (48.5%)	74 (52.1%)	29 (53.7%)	
High school or more	44 (33.8%)	25 (17.6%)	12 (22.2%)	
Living room (% shared)	104 (80.0%)	115 (81.0%)	50 (92.6%)	χ^2^ = 4.60, *p* = 0.100
Length of stay				χ^2^ = 2.57, *p* = 0.632
≤ 1 year	56 (43.1%)	49 (34.5%)	20 (37.0%)	
1–3 years	44 (33.8%)	54 (38.0%)	18 (33.3%)	
> 3 years	30 (23.1%)	39 (27.5%)	16 (29.6%)	
**Health characteristics**				
Pain (% Yes)	44 (33.8%)	70 (49.3%)	29 (53.7%)	χ^2^ = 9.12, *p* = 0.010 (1 < 2 and 3)
IADLs (% dependent)	71 (54.6%)	99 (69.7%)	29 (53.7%)	χ^2^ = 7.97, *p* = 0.018 (1 and 3 < 2)
ADLs (% dependent)	87 (66.9%)	111 (78.2%)	35 (64.8%)	χ^2^ = 5.62, *p* = 0.060
FCI (median and IQR)	1.00 (2.25)^#^	2.00 (2.00)^#^	2 (2.00)^#^	KW = 10.10, *p* = 0.006 (1 < 2 and 3)
MMSE	20.99 ± 5.21	21.54 ± 5.21	22.76 ± 4.44	F = 2.30, *p* = 0.102

Notes: 1) C1, good sleepers; C2, poor sleepers without hypnotic use; and 3) C3, poor sleepers with hypnotic use.

2) PSQI: Pittsburgh Sleep Quality Index; FCI, Functional Comorbidity Index; ADLs, Activities of Daily Living; IADLs, Instrumental Activities of Daily Living; MMSE, Mini-Mental State Examination.

3) ^#^: median and interquartile range.

### The differences of participants’ characteristics across sleep patterns

The comparisons concerning residents’ demographic and health characteristics across the sleep patterns were demonstrated in Table 3. Residents’ gender, education, FCI, pain, and IADLs significantly differed among the identified sleep patterns. Specifically, compared with ‘good sleepers’, residents in the other two patterns (poor sleepers) were likely to experience more pain and more chronic conditions (*p* < 0.05). Among the three patterns, the ‘poor sleepers without hypnotic use’ had the highest percentage of dependence on IADLs, while the ‘poor sleepers with hypnotic use’ had the highest proportion of females (*p* < 0.05). Besides, the ‘poor sleepers without hypnotic use’ had a lower level of education than the ‘good sleepers’ (*p* < 0.05). The remaining demographic and health characteristics did not show statistical differences across the three sleep patterns, despite the ‘poor sleepers with hypnotic use’ having the highest average age, the highest mean score of MMSE, the highest percentages of residents living in shared rooms, and staying at nursing homes for > 3 years, and the lowest proportion of dependent residents in ADLs.

### The associations of mental health with sleep patterns

The relationships between sleep patterns and residents’ mental health are shown in Table 4 and Table S1. The unadjusted logistic regression models suggested that ‘poor sleepers without hypnotic use’ (OR = 3.73, 95% CI = 2.09, 6.65, *p* < 0.001) and ‘poor sleepers with hypnotic use’ (OR = 5.24, 95% CI = 2.54, 10.79, *p* < 0.001) were more likely to have depressive symptoms relative to ‘good sleepers’. And there was no significant difference in depressive symptoms between the two groups of poor sleepers (OR = 1.41, 95% CI = 0.72, 2.74, *p* = 0.319). In terms of anxiety symptoms, compared with ‘good sleepers’, ‘poor sleepers with hypnotic use’ were significantly more likely to have anxiety symptoms (OR = 5.02, 95% CI = 2.13, 11.83, *p* < 0.001), but ‘poor sleepers without hypnotic use’ not (OR = 2.04, 95% CI = 0.97, 4.29, *p* = 0.062). Moreover, ‘poor sleepers with hypnotic use’ were associated with more anxiety symptoms than the ‘poor sleepers without hypnotic use’ (OR = 2.47, 95% CI = 1.15, 5.30, *p* = 0.021). After adjusting the resident- and facility-level covariates, the above relationships remained unchanged.

**Table 4:** The associations of mental health with sleep patterns

	Depressive symptoms			Anxiety symptoms	
	Odds Ratio (95% CI)	P		Odds Ratio (95% CI)	P
Unadjusted					
C2 vs. C1^a^	3.73 (2.09, 6.65)	< 0.001		2.04 (0.97, 4.29)	0.062
C3 vs. C1^a^	5.24 (2.54, 10.79)	< 0.001		5.02 (2.13, 11.83)	< 0.001
C3 vs. C2^b^	1.41 (0.72, 2.74)	0.319		2.47 (1.15, 5.30)	0.021
Adjusted^*^					
C2 vs. C1^a^	3.12 (1.65, 5.93)	< 0.001		1.37 (0.61, 3.08)	0.442
C3 vs. C1^a^	4.24 (1.92, 9.34)	< 0.001		4.37 (1.69, 11.32)	0.002
C3 vs. C2^b^	1.36 (0.65, 2.82)	0.413		3.18 (1.33, 7.58)	0.009

Notes: 1) ^a^: C1 was reference group, ^b^: C2 was reference group;

2) C1: ‘good sleepers’ pattern, C2: ‘poor sleepers without hypnotic use’ pattern, C3: ‘poor sleepers with hypnotic use’ pattern;

3) ^*^: Covariates included resident- (i.e., gender, age, education, living room, length of stay, pain, ADLs, IADLs, FCI, and MMSE) and facility-level variables (i.e., ownership, size, affiliation, occupation rate, staff-resident ratio, staff working hours per day, and outdoor sites).

## Discussion

Using the seven sleep dimensions from a well-established instrument in a sample size of nursing home residents, we identified three distinct sleep patterns (i.e., ‘good sleepers’, ‘poor sleepers without hypnotic use’, and ‘poorer sleepers with hypnotic use’) via LPA. Compared with poor sleepers in Class 2 and Class 3, good sleepers in Class 1 reported better overall sleep quality including shorter latency, longer duration, higher efficiency and subjective quality, fewer disturbances, and less daytime dysfunction. Poor sleepers in Class 2 and Class 3 reported quite similar sleep issues except for the use of sleep medication. ‘Poor sleepers with hypnotic use’ in Class 3 reported the highest level of sleep medication use. The findings suggested significant associations with residents’ mental health. That is, in comparison with ‘good sleepers’, ‘poor sleepers with hypnotic use’ had more depressive and anxiety symptoms, and ‘poor sleepers without hypnotic use’ had more depressive symptoms. To our best knowledge, this is the first study that determines the clinically meaningful sleep patterns for residents in nursing homes. The findings of this study support the distinction in sleep patterns among nursing home residents, and motivate future studies to develop effective and tailored intervention strategies to facilitate the management of residents’ sleep and mental health via targeting at the potentially modifiable factors in sleep.

The present findings of three sleep patterns (‘good sleepers’, ‘poor sleepers without hypnotic use’, and ‘poorer sleepers with hypnotic use’) in nursing home residents were partially consistent with those reported in previous studies conducted on older adults [4, 7, 8]. The pattern of ‘good sleepers’ was similarly identified in Leigh et al. (2015) study [7] as ‘untroubled sleepers’, presenting few problems in all sleep dimensions. Despite previous literature having suggested the existence of poor sleep patterns characterized by a variety of sleep problems, such as the ‘multiple problems’ group in Yu et al. (2017) study [4] and ‘trouble sleepers’ in Leigh et al. (2015) study [7], our findings indicated that sleep medication use was an important dimension that distinguishes the patterns of poor sleep. The different presentations in sleep medication use can be explained by older adults’ various opinions on using hypnotics for improving sleep [36]. Some older adults preferred to tolerate symptoms of poor sleep and not use the prescribed sleep medications, because they regarded poor sleep as one of the consequences of normal aging and held negative beliefs about using hypnotics due to the potential risks of dependency and withdrawal complications [36]. On the other hand, studies have suggested that older individuals probably took hypnotic drugs in an inappropriate manner, such as being reluctant to take prescribed drugs or continuous use without any pause, which might therefore minimize the treatment effects and not improve their sleep quality [37, 38].

Besides, the present study did not find any patterns solely characterized by a single sleep problem, like ‘trouble falling asleep’ in Leigh et al. (2015) study [7], or ‘inadequate sleep’ (low sleep duration) and ‘disturbed sleep’ (reporting problem in sleep disturbance) in Yu et al. (2017) study [4]. The co-existence of multiple sleep problems among nursing home residents might be mainly ascribed to residents’ poor sleep hygiene (e.g., daytime inactivity, long time spent in bed) and the facility’s complex living environments (e.g., nighttime bedroom light and noise) [1, 39].

In terms of the difference in residents’ characteristics across the sleep patterns, the findings of this study suggested that relative to ‘good sleepers’, ‘poor sleepers with hypnotic use’ were more likely to be female; ‘poor sleepers without hypnotic use’ were more likely to be less educated and dependent on IADLs; and both ‘poor sleepers’ groups experienced more pain and chronic conditions. These results of residents’ characteristics correspond with previous studies that revealed the significantly correlated factors of sleep in older adults [1, 40–44]. The reasons for more females in ‘poor sleepers with hypnotic use’ versus ‘good sleepers’ could be considered from the biological differences (e.g., hormonal modulation) and behavioral factors (e.g., more rumination behaviors and sensitive emotions increasing susceptibility of sleep problems) [40, 45, 46]. For more residents with dependent IADLs in ‘poor sleepers without hypnotic use’, it might be that they had less autonomy and capability of independently implementing structured social-oriented activities, such as medicine intake in an appropriate manner [10]. The results of more chronic conditions and pain in residents with poor sleep patterns might be related to their physical symptoms (e.g., shortness of breath), disrupted sleep continuity and decreased sleep efficiency [43, 47, 48]. Recognizing residents’ different characteristics across sleep patterns may help differentiate residents with distinct sleep patterns and design targeted and individualized intervention strategies. Given that few studies have investigated the sleep patterns among nursing home residents to date, more research is warranted to better understand how the different sleep patterns vary on a broader range of characteristics in such a population.

Regarding the associations with mental health, this study indicated that compared with ‘good sleepers’, residents with poor sleep, regardless of using hypnotics, were more likely to have depressive symptoms, whereas only poor sleepers using hypnotics had significantly more anxiety symptoms. Previous studies have also proved the significant relationships between older adults’ sleep patterns and their mental health [4, 8]. For instance, older adults with ‘multiple problems’ sleep patterns had significantly higher levels of depression and anxiety relative to those without sleep problems [4]. The potential explanations for poor sleep corresponding to greater depressive symptoms can be considered from the perspectives of physiological mechanisms (e.g., inflammation hypothesis, biochemical pathways, and circadian rhythm) and psychosocial manners [49, 50].

In addition, the findings of this study indicated that the poor sleep patterns in nursing home residents could be significantly associated with different levels of anxiety symptoms due to the difference in using hypnotics. As suggested by the previous study, the use of hypnotics was significantly associated with a higher risk of severe anxiety symptoms [38]. One possible explanation for the occurrence of anxiety symptoms might be that older individuals were concerned about the side effects, misuse, and withdrawal effects caused by using hypnotics [37]. Moreover, the lack of reliable information on the pros and cons of using hypnotics and the vague intake instructions (e.g., ‘when required’) might induce their uncertainty about the consequences to themselves, thereby exacerbating their anxiety symptoms [37]. Another possible explanation is that residents who were more anxious were given hypnotics by nursing home clinicians. Some sleep-promoting medications (e.g., benzodiazepine) are not only effective for insomnia but also prescribed by clinicians to treat anxiety disorders [51]. The findings indicate the possibility of improving residents’ mental health by designing interventions with considering the individual differences in sleep patterns, e.g., in terms of whether or not using hypnotics.

The findings of this study provide some implications for clinical practice among nursing home residents. This study suggested the distinction in sleep patterns among nursing home residents, which indicates the possibility and necessity for future research to adopt tailored intervention strategies, instead of a one-size-fits-all approach, to achieve better improvements in residents’ sleep. Specifically, a comprehensive assessment of residents’ health conditions and needs is essential before developing tailored sleep interventions. For those who experience poor sleep but do not take sleep medications, nursing care clinicians can provide individualized medication administration based on their health conditions and needs. As the American Academy of Sleep Medicine recommends, sleep medication can be reasonably utilized when necessary (e.g., for those screened for contraindications) by considering individuals’ preferences and carefully monitoring potential risks [52]. For those ‘poor sleepers’ who take sleep medications, educational interventions can be provided to increase their awareness and modify their behaviors in medication intake [53]. In addition to pharmacological treatment, psychological treatment, such as cognitive behavioral therapy [15, 54] and mindfulness-based intervention [55], may be applicable and effective for those who experience both poor sleep and psychological symptoms. Besides, non-pharmacological interventions such as sleep hygiene education or environmental adjustments (e.g., light exposure therapy) can be considered to further improve sleep quality and promote mental health if needed [56, 57].

### Limitations

The findings of this study are subject to several limitations. First, this study may be subject to selection bias. Nursing home residents are sicker and more medically complex than those living in the community. It is possible that they would report poor sleep even when they lived in their home. The high prevalence of poor sleep (62.6%) reported by nursing home residents in our sample indicated that both residents’ health status and facility environmental factors played a significant role in sleep quality. Future research is needed to identify the magnitude of environmental factors’ influence on sleep disturbance among nursing home residents. Moreover, convenience sampling was used to recruit nursing home residents, which might limit the generalizability of our findings. All nursing homes in this study were located in urban areas and residents with severe cognitive impairment were not enrolled. Our findings on sleep patterns and their relationships with mental health cannot be generalized to residents with severe cognitive impairment and those living in rural nursing facilities. However, as the study sample shares similar characteristics (e.g., age, functional status, and depressive symptoms) with residents from other national nursing home studies in China [58–60], the study findings may be applicable to nursing home residents outside of Jinan. Second, the cross-sectional study design prohibited the ability to examine longitudinal sleep variability, or to determine the causal relationships and potential mechanisms between sleep patterns and mental health. Therefore, future work can be conducted to replicate these findings in a larger-scale and longitudinal cohort by adding objective measures (e.g., actigraphy) to comprehensively capture the sleep patterns and by including residents with severe cognitive impairment.

## Conclusions

This study revealed three distinct sleep patterns among nursing home residents and suggested the different demographic and health characteristics across these sleep patterns. The findings indicated that compared with good sleepers, residents with poor sleep, with or without hypnotic use, were more likely to have depressive symptoms; whereas only poor sleepers using hypnotics had more anxiety symptoms. These findings may guide future research on designing intervention strategies tailored to residents with distinct sleep patterns, which may promote desirable improvements in sleep and mental health for nursing home residents.

### Electronic supplementary material

Below is the link to the electronic supplementary material.

Supplementary Material 1: Table S1 The associations of sleep patterns with mental health (adjusted model)

## Data Availability

The data that support the findings of this study are available on request from the corresponding author. The data are not publicly available due to privacy or ethical considerations.

## Abbreviations

ADLs: activities of daily living
AIC: Akaike’s Information Criterion
BIC: Bayesian Information Criterion
BLRT: Bootstrapped-Likelihood Ratio Test
CIs: confidence intervals
FCI: Functional Comorbidity Index
GAD-2: Generalized Anxiety Disorder Scale-2
IADLs: instrumental activities of daily living
LMRT: Lo-Mendell-Rubin Likelihood Ratio Test
LPA: Latent Profile Analysis
MMSE: Mini-Mental State Examination
ORs: odds ratios
PHQ-9: Patient Health Questionnaire-9
PSQI: Pittsburgh Sleep Quality Index
