# Supplementary material for: The sleep patterns and their associations with mental health among nursing home residents: a latent profile approach
Source: BMC Geriatr. 2023 Aug 3;23:468. doi: 10.1186/s12877-023-04124-5 (PMC10401828; doi:10.1186/s12877-023-04124-5)
Supplement: Supplementary file 1 — Supplementary Material 1: Table S1 The associations of sleep patterns with mental health (adjusted model) [file 12877_2023_4124_MOESM1_ESM.docx]

Table S1. The associations of sleep patterns with mental health (adjusted model)

|  | Depression | | |  | Anxiety | | |
| --- | --- | --- | --- | --- | --- | --- | --- |
|  | Coefficient | *p* | Odds Ratio (95% CI) |  | Coefficient | *p* | Odds Ratio (95% CI) |
| Sleep (ref. C1) |  |  |  |  |  |  |  |
| C2 | 1.14 | <0.001 | 3.12 (1.65, 5.93) |  | 0.32 | 0.442 | 1.37 (0.61, 3.08) |
| C3 | 1.44 | <0.001 | 4.24 (1.92, 9.34) |  | 1.47 | 0.002 | 4.37 (1.69, 11.32) |
| Gender (ref. male) | 0.04 | 0.890 | 1.04 (0.56, 1.94) |  | -0.42 | 0.321 | 0.66 (0.29, 1.51) |
| Age | -0.01 | 0.725 | 0.99 (0.96, 1.03) |  | -0.01 | 0.556 | 0.99 (0.94, 1.03) |
| Education (ref. illiterate) |  |  |  |  |  |  |  |
| Elementary/middle school | -0.27 | 0.461 | 0.76 (0.37, 1.58) |  | -1.07 | 0.022 | 0.34 (0.14, 0.86) |
| High school or more | -0.04 | 0.925 | 0.96 (0.37, 2.44) |  | -1.86 | 0.005 | 0.16 (0.04, 0.57) |
| Living room (ref. single/couple) | 0.83 | 0.045 | 2.29 (1.02, 5.14) |  | 1.28 | 0.057 | 3.59 (0.96, 13.40) |
| Length of stay (ref. ≤ 1 year) |  |  |  |  |  |  |  |
| 1-3 years | -0.14 | 0.653 | 0.87 (0.46, 1.62) |  | -0.71 | 0.102 | 0.49 (0.21, 1.15) |
| >3 years | 0.12 | 0.714 | 1.13 (0.59, 2.16) |  | 0.33 | 0.402 | 1.39 (0.65, 2.97) |
| Pain (ref. no) | 0.88 | 0.002 | 2.42 (1.39, 4.20) |  | 1.11 | 0.002 | 3.04 (1.49, 6.18) |
| FCI | 0.28 | 0.194 | 1.33 (0.87, 2.04) |  | 0.04 | 0.877 | 1.04 (0.61, 1.78) |
| ADLs (ref. independent) | 0.63 | 0.064 | 1.88 (0.96, 3.68) |  | 0.57 | 0.230 | 1.77 (0.70, 4.50) |
| IADLs (ref. independent) | -0.03 | 0.928 | 0.97 (0.52, 1.81) |  | 0.51 | 0.239 | 1.66 (0.71, 3.85) |
| MMSE | 0.01 | 0.781 | 1.01 (0.95, 1.08) |  | 0.02 | 0.718 | 1.02 (0.93, 1.11) |
| Ownership (ref. not-for-profit) | -0.04 | 0.960 | 0.96 (0.23, 3.98) |  | -0.55 | 0.599 | 0.58 (0.07, 4.49) |
| Size (total beds) | -0.32 | 0.677 | 0.72 (0.16, 3.32) |  | 0.99 | 0.280 | 2.69 (0.45, 16.25) |
| Affiliation (ref. freestanding) | -0.99 | 0.192 | 0.37 (0.08, 1.64) |  | -1.33 | 0.269 | 0.26 (0.03, 2.79) |
| Occupation rate | 0.44 | 0.664 | 1.55 (0.21, 11.27) |  | 0.24 | 0.826 | 1.27 (0.15, 10.94) |
| Staff-resident ratio | -0.14 | 0.152 | 0.87 (0.72, 1.05) |  | 0.08 | 0.416 | 1.08 (0.89, 1.32) |
| Staff working hours (per week) | -0.20 | 0.270 | 0.82 (0.58, 1.16) |  | -0.22 | 0.379 | 0.80 (0.49, 1.31) |
| Outdoor sites (ref. no) | -0.69 | 0.100 | 0.50 (0.22, 1.14) |  | -0.09 | 0.849 | 0.91 (0.36, 2.32) |
| Note: 1) C1: good sleepers; C2: poor sleepers without hypnotic use; 3) C3: poor sleepers with hypnotic use;  2) IADLs, Instrumental Activities of Daily Living; FCI, Functional Comorbidity Index; ADLs, Activities of Daily Living; MMSE, Mini-Mental State Examination. | | | | | | | |
